# The Prebiotic and Probiotic Properties of Human Milk: Implications for Infant Immune Development and Pediatric Asthma

**DOI:** 10.3389/fped.2018.00197

**Published:** 2018-07-24

**Authors:** Shirin Moossavi, Kozeta Miliku, Shadi Sepehri, Ehsan Khafipour, Meghan B. Azad

**Affiliations:** ^1^Department of Medical Microbiology and Infectious Diseases, University of Manitoba, Winnipeg, MB, Canada; ^2^Children's Hospital Research Institute of Manitoba, Winnipeg, MB, Canada; ^3^Developmental Origins of Chronic Diseases in Children Network (DEVOTION), Children's Hospital Research Institute of Manitoba, Winnipeg, MB, Canada; ^4^Digestive Oncology Research Center, Digestive Disease Research Institute, Tehran University of Medical Sciences, Tehran, Iran; ^5^Pediatrics and Child Health, University of Manitoba, Winnipeg, MB, Canada; ^6^Department of Animal Science, University of Manitoba, Winnipeg, MB, Canada

**Keywords:** human milk, microbiota, human milk oligosaccharides, immune development, asthma, pediatrics, probiotic, prebiotic

## Abstract

The incidence of pediatric asthma has increased substantially in recent decades, reaching a worldwide prevalence of 14%. This rapid increase may be attributed to the loss of “Old Friend” microbes from the human microbiota resulting in a less diverse and “dysbiotic” gut microbiota, which fails to optimally stimulate immune development during infancy. This hypothesis is supported by observations that the gut microbiota is different in infants who develop asthma later in life compared to those who remain healthy. Thus, early life exposures that influence gut microbiota play a crucial role in asthma development. Breastfeeding is one such exposure; it is generally considered protective against pediatric asthma, although conflicting results have been reported, potentially due to variations in milk composition between individuals and across populations. Human milk oligosaccharides (HMOs) and milk microbiota are two major milk components that influence the infant gut microbiota and hence, development of the immune system. Among their many immunomodulatory functions, HMOs exert a selective pressure within the infant gut microbial niche, preferentially promoting the proliferation of specific bacteria including *Bifidobacteria*. Milk is also a source of viable bacteria originating from the maternal gut and infant oral cavity. As such, breastmilk has prebiotic and probiotic properties that can modulate two of the main forces controlling the gut microbial community assembly, i.e., dispersal and selection. Here, we review the latest evidence, mechanisms and hypotheses for the synergistic and/or additive effects of milk microbiota and HMOs in protecting against pediatric asthma.

## Breastfeeding, asthma, and the microbiota

Breastfeeding has many established benefits for maternal and child health (1), including a potentially protective effect against pediatric asthma development. In a meta-analysis of 117 studies, Dogaru et al. found that breastfeeding was associated with a 22% reduced risk of asthma, with the strongest effects observed during early childhood (2). This association was not seen among adults in the population-based UK Biobank study (3); however, breastfeeding data were self-reported and did not account for duration or exclusivity. Several plausible mechanisms have been proposed to explain how breastfeeding might protect against asthma (4). For example, breastfeeding appears to mitigate the harmful effects of asthmogenic exposures including air pollution (5) and psychosocial stress (6). In addition, breastfeeding has been shown to support lung growth (7) and enhance lung function (8). Recently in the Canadian Healthy Infant Longitudinal Development (CHILD) Study, we reported a dose-dependent reduced risk of wheezing (9) and asthma (10) among breastfed children. These associations were stronger among infants fed at the breast compared to those receiving pumped breast milk, although both were superior to infant formula (10). This suggests that the act of suckling and/or skin-to-skin contact may contribute to the protective effect of breastfeeding. Alternatively or in addition, this finding could reflect a role for bioactive components in human milk, which may be altered during the pumping and storage process.

Breastfeeding also profoundly influences development of the infant oral and gut microbiota (11), which have been independently linked with asthma development (12, 13). Recent increases in asthma prevalence (14) have been attributed to a loss of diversity within the human microbiota due to the dramatic lifestyle changes in the last century (15). “Old Friend” and “missing microbe” hypotheses speculate that our increasingly hygienic lifestyle and overuse of antibiotics have resulted in the loss of specific bacteria from the modern day human microbiota (15, 16). Given the co-adaptation and co-evolution of the “ancient” microbiota with the human immune system, it is plausible that their loss could result in aberrant immune responses leading to allergic, autoimmune, and inflammatory diseases, including asthma (16). This hypothesis is supported by evidence that skin and nasal microbiota differ between adjacent but socioeconomically contrasting regions of Finnish and Russian Karelia with discordant prevalence of asthma and allergy (17). Consistent with this line of evidence, gut microbiota profiles have also been shown to differ between infants who do or do not develop asthma in the CHILD cohort and other longitudinal studies (12, 13). In addition, early life exposures that alter the microbiota (such as antibiotic use, cesarean delivery, and formula feeding) have been linked to asthma development (18).

**Figure 1:**
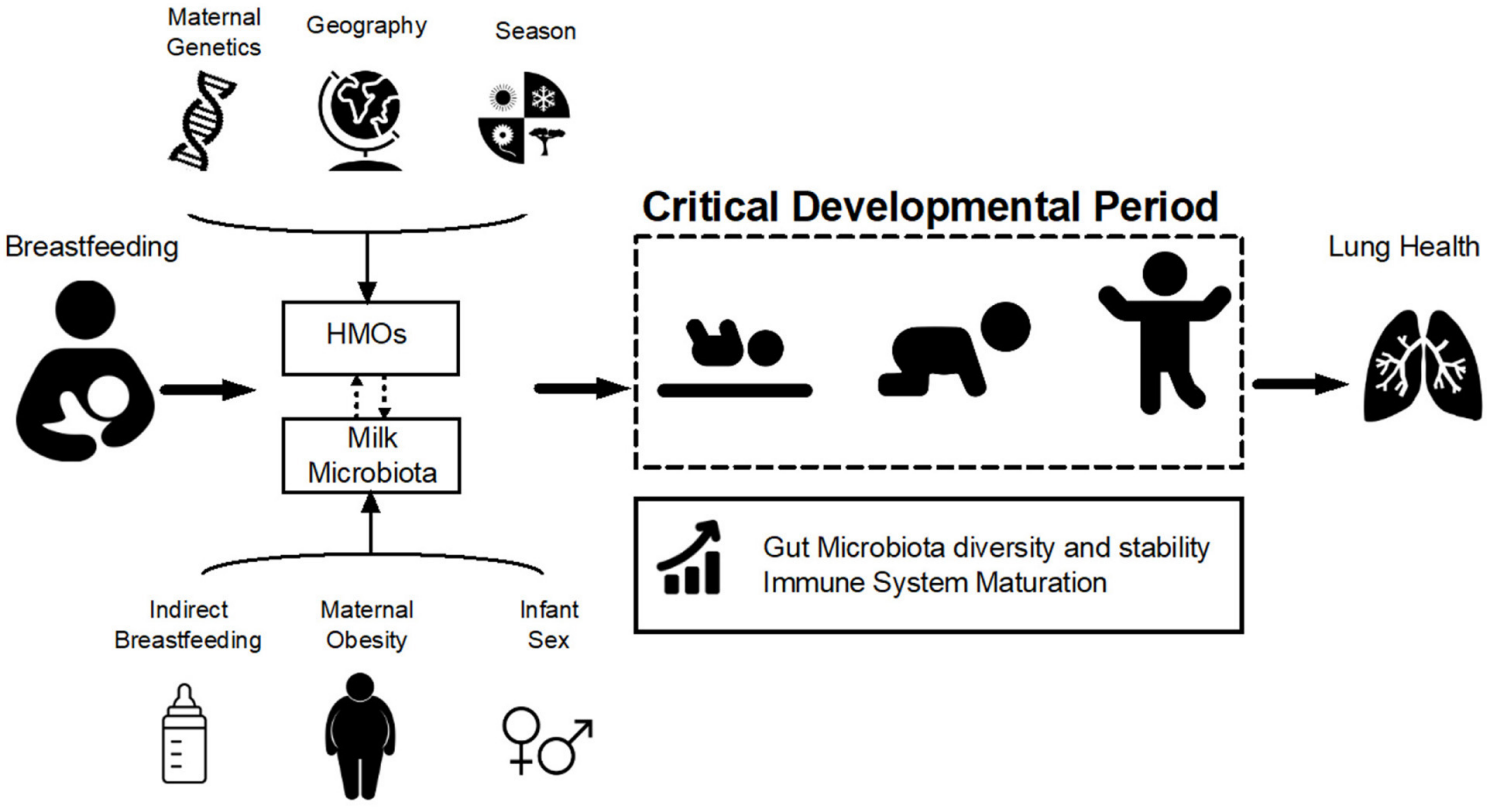
Hypothesized pathways of association between breastfeeding and lung health. Breastfeeding influences infant gut microbiota development and stability during the critical developmental period in early life via two of its main components: human milk oligosaccharides (HMOs) and milk microbiota. HMOs exert a selective pressure within the infant gut microbial niche, preferentially promoting the proliferation of specific bacteria including *Bifidobacteria*. Milk is also a source of viable bacteria originating from the maternal gut and infant oral cavity. HMO composition is influenced by maternal genetics, geography, and season while microbiota is affected by maternal weight status, mode of breastfeeding and infant sex (Table 1). Variations in HMOs and milk microbiota could modulate the effect of breastfeeding on the infant gut microbiota, which in turn shapes the infant immune system and could ultimately influence lung health and asthma development.

Human milk oligosaccharides (HMOs) and microbiota are two major milk components that influence infant gut microbiota and hence, development of the immune system. As such, breastmilk has prebiotic and probiotic properties that can modulate two of the main forces controlling the gut microbial community assembly, i.e., dispersal (acquiring new bacterial species) and selection (achieving a permissive environment to facilitate sustainable colonization). If these processes are disrupted, the infant gut microbiota developmental trajectory will be altered, potentially leading to a suboptimal final composition. This could be one of the underlying mechanisms of predisposition to a range of chronic diseases including allergy and asthma (19) (Figure 1). Here, we review the latest evidence and hypothesized mechanisms for the synergistic and/or additive effects of HMOs and milk microbiota in protecting against pediatric asthma.

## Prebiotic and probiotic properties of human milk

Breastfeeding affects both gut microbiota and immune system development (20, 21). Human milk functions as a bioactive food consisting of all essential nutrients plus immune components, hormones, HMOs, and microbiota, which serve crucial roles in early life metabolic and immune system homeostasis and development (22). HMOs and the microbiota are of particular interest because of their influence on the infant gut microbiota and potential long-term health importance (22) (Table 1).

**Table 1:** Evidence on factors influencing the composition of human milk oligosaccharides (HMOs) and milk microbiota and their association with pediatric asthma.

**Milk components**	**Influenced by**	**Impact on gut microbiota**	**Association with asthma**
Human milk oligosaccharides	Host genetics (23, 24) Geography (24, 25) Season (26, 27) Parity (26) Lactation stage (28) Birth mode (29)	Influence nutrient availability for gut bacteria (30) Enrich *Bacteroides* and *Bifidobacterium* spp. (30) Prevent pathogen colonization (31) Modify host-microbe interaction (32)	No published evidence, but associations found for atopic sensitization and food allergy (26, 33)
Milk microbiota	Birth mode (22) Mode of breastfeeding (22) Maternal BMI (34) Infant sex (34)	Provide pioneering species (11) Enrich “beneficial” bacteria (35) Increase colonization efficiency	No published evidence

### Human milk oligosaccharides

HMOs constitute the third largest component of human milk (31). These structurally diverse carbohydrates are synthesized by sequential addition of monosaccharides to lactose, and various α-glycosidic linkages of fucose and/or sialic acid to the core molecules (23, 36). More than 100 different HMOs have been identified, with the amount and composition varying substantially between women and over the course of lactation (25, 28, 37, 38). HMO fucosylation is regulated by enzymes encoded by the fucosyltransferase 2 (FUT2) and FUT3 genes, which determine secretor status and Lewis blood group status, respectively (23, 24). However, geographical variation in HMO composition suggests that non-genetic factors such as sociocultural and environmental factors may also play a role (24, 25). In the CHILD cohort we have observed that, beyond genetic FUT2 secretor status, HMO composition is associated with ethnicity, lactation stage, parity, geographic location, season of collection, and breastfeeding exclusivity (26).

Although still an emerging field of research, many biological functions have been attributed to HMOs (24, 31). The majority of ingested HMOs reach the lower intestinal tract where they can function as prebiotics, providing selective substrates for gut bacteria (30), as discussed below. Preliminary evidence also suggests that specific HMOs could directly modulate the immune response, with studies in pigs (39) and mice (40, 41) demonstrating direct effects on viral pathogens as well as host immune cells. In breastfed infants, approximately 1% of HMOs are absorbed into the peripheral circulation, potentially reaching all organs including the lungs (31), thus it is plausible that HMOs could affect lung mucosal immunity by interacting with airway epithelia, immune cells, potential pathogens or resident microbes, providing another mechanism for protecting against asthma.

While the mechanisms are not fully elucidated, there is some evidence that supplementation with prebiotics (42, 43) or HMOs (44) may be protective against allergy and asthma in animal models and human studies. A recent systematic review reported a reduction in asthma among high-risk infants given prebiotics, such as galacto-oligosaccharide (GOS) and fructo-oligosaccharide (FOS) (45). However, GOS and FOS are structurally distinct from HMOs, which have not been widely studied in relation to asthma, although a few studies have examined their association with allergic disease. One study found that infants receiving milk with low Lacto-N-fucopentaose III concentrations were more likely to develop cow's milk allergy (33), an effect that might be modulated by birth mode (29). In the CHILD cohort, we have observed that HMO composition (but not any individual HMO) is associated with the development of allergic sensitization during infancy (26). Associations with asthma during early childhood are currently being explored. Altogether, epidemiological and experimental studies support the prebiotic effects of HMOs and suggest a potential role in asthma development (Table 1), but further research is needed to confirm and characterize this relationship.

### Milk microbiota

Culture-dependent and independent studies have confirmed the presence of bacteria in human milk (46). It is estimated that breastfed infants receive 10^4^-10^6^ bacteria per day (based on an average daily consumption of 800 mL of milk) with most isolated species belonging to the genera *Staphylococcus, Streptococcus, Lactobacillus*, and *Bifidobacterium* (47). Culture-independent (DNA sequencing-based) approaches in the CHILD cohort (34) and others (48) have recovered a higher diversity of bacteria in breast milk including lactic acid bacteria (*Enterococcus* and *Lactococcus*), oral-derived (*Veillonella* and *Gemella*), skin-associated (*Cutibacterium* and *Staphylococcus*), and environmental bacteria (*Pseudomonas* and *Sphingomonas*) with a high degree of inter-individual variability. While bacterial load appears to remain constant during milk maturation (49), it gradually decreases during one feed (50).

The milk microbiota is suggested to originate from the maternal gut, breast tissue, or infant oral cavity (51). Depending on the source of bacteria, different factors may contribute to shaping the milk microbiota (Table 1). For example, while maternal factors could influence the mother's gut bacteria, early life factors such as mode of delivery and mode of breastfeeding (directly at the breast vs. expressed and bottled breast milk) could potentially alter the exogenous bacteria derived from the infant (22). In the CHILD cohort, we have found that mode of breastfeeding was significantly associated with milk microbiota composition, with expressed milk feeding favoring depletion of *Bifidobacteria* and enrichment with potential pathogens and environmental bacteria (34). We also observed some sex-specific associations (e.g., maternal BMI associated with milk microbiota only if the infant is female) while other factors demonstrated a phylum-specific effect (e.g., maternal atopy associated with Actinobacteria richness) (34). As discussed below, the milk microbiota is suggested to provide a mechanism of vertical microbial transmission from the mother to the infant.

To the best of our knowledge, no study to date has directly linked milk microbiota with pediatric asthma and allergy. However, we have previously reported that indirect breastfeeding was associated with higher risk of pediatric asthma in the CHILD study (10), and we have also observed that mode of breastfeeding is consistently associated with milk microbiota composition in this cohort (34). It is plausible that the milk microbiota could play a role in asthma development, conceivably via the modulation of gut microbiota. Studies to date have been inconclusive regarding the use of commercial probiotics for preventing asthma (52); however, the natural milk microbiota is a complex community, and hence, may be more effective in this regard, as it has presumably evolved to optimally support infant gut microbiota and immune development.

## Contribution of HMOs and milk microbiota to infant gut microbiota development

Dietary exposures are among the most influential factors shaping the infant gut microbiota. Breastmilk not only provides nutrients to the infant, but is also a source of probiotics (milk microbiota) and prebiotics (HMOs) contributing to the establishment of the infant gut microbiota (11). The human gut microbiota develops through a complex process of stepwise successions beginning at birth (53). First colonizers of the infant gut are facultative anaerobes, including the *Staphylococcus, Streptococcus*, and *Enterococcus* genera, and the Enterobacteriaceae family. These pioneering bacteria reduce the redox potential and hence, create a favorable condition for the growth of obligate anaerobes such as the *Bifidobacterium, Bacteroides, Clostridium*, and *Eubacterium* genera (53). In contrast to the mature gut microbiota, the infant gut is dominated by *Bifidobacterium* species prior to weaning, including *B. longum, B. breve*, and *B. bifidum* (35, 54). HMOs are specifically utilized by *Bacteroides* and *Bifidobacterium* spp. (30) leading to dominance of these taxa in breastfed infants (55), with different *Bifidobacterium* species and strains demonstrating different preferences for specific HMOs (56). Furthermore, rates of absorption and excretion are different for different categories of HMOs (57), resulting in different amounts reaching the colon; further highlighting the nuanced impact of the HMO composition on the infant gut microbiota. HMOs could also indirectly affect the gut microbiota composition through their decoy activity that prevents pathogen colonization (31). Additionally, HMOs can modify host-microbe interactions by affecting epithelial cell turnover (32) and glycocalyx mucus formation (58).

In addition to the prebiotic role of HMOs, the viability of milk bacteria suggests that human milk could function as a probiotic shaping the infant gut microbiota by facilitating dispersal (22). Milk microbiota may provide pioneering species and impact the final gut microbiota composition (59). Human microbiota studies demonstrating strain similarities between maternal gut, milk, and infant gut support this hypothesis (54), and find that *Bifidobacterium* spp. constitute the majority of shared taxa between maternal milk and infant stool (35). It is plausible that human milk specifically enriches and protects “beneficial” bacteria as they are transported through the infant's acidic stomach environment to the lower intestinal tract. Milk could also function as an incubator of bacteria to increase their “dose,” thereby increasing the likelihood of successful colonization in the infant gut (60).

## Infant gut microbiota, immune system development, and asthma

The precise role of milk microbiota and HMOs in shaping the infant gut microbiota and immune development is an active area of research with potential implications for asthma prevention. The gastrointestinal tract is the largest immune organ in the human body and it plays a crucial role in the education and subsequent maturation of immune system by (i) maintaining immunological tolerance to food components as well as the commensal microbiota, and (ii) acquiring the capacity to appropriately respond to pathogenic microbes (61, 62). Moreover, the immune system is essential in keeping the balance of bacterial communities and preventing dysbiosis (61, 63). This host-microbiota crosstalk is shaped early in life (63, 64). It has been hypothesized and experimentally demonstrated that alteration of the early gut microbiota can disrupt the microbially-mediated mechanisms of immunological tolerance, resulting in predisposition to allergic disorders including asthma (61, 65).

Development of the immune system begins in utero, and continues during infancy (18). At birth, the adaptive immune system is dominated by the T-helper cell type 2 (Th2) which will later shift to Th1 and Th17 phenotype (66, 67). Delayed or impaired Th2/Th1 transition during early infancy is associated with increased risk of atopic disorders, including asthma (18). It has been shown that the gut microbiota stimulates regulatory T cells that help monitor the Th1/Th2 balance (18, 61, 62). Furthermore, exposure to microbes or microbial products such as microbe-associated molecular patterns (MAMPs) or short chain fatty acids (SCFA) during the early infancy is essential for stimulating the infant's naïve and immature immune system (53, 67), gut-associated lymphoid tissue development (64, 66), and B cell maturation (68). Thus, early life microbiota disruption could lead to alterations in several immune parameters including regulatory T cells proliferation, Th17 response, and IgE response (53), all of which contribute to asthma development.

A growing body of evidence supports the role of gut microbiota in the development of allergic disorders (64, 69, 70). However, it is not clear whether the risk of asthma development is modulated by presence of specific bacteria, temporal succession patterns, or the overall taxonomic and functional diversity of the microbial community (53). Observational human studies have indicated that lower gut microbiota diversity early in life could be associated with higher risk of asthma at later time points (12). In contrast, gut microbiota diversity and overall community composition were not associated with atopic wheezing in the CHILD cohort, although lower relative abundances of specific bacterial genera including *Faecalibacterium, Lachnospira, Veillonella*, and *Rothia* were associated with this phenotype (13). These genera were further shown to reduce clinical features of asthma including respiratory tract neutrophil and lymphocyte infiltrations and histopathological alterations in a mouse model of ovalbumin-induced asthma (13). Similarly, other studies have found that higher relative abundances of specific taxa including *Clostridium difficile* and *Clostridium neonatale* in the infant gut microbiota are associated with an increased risk of asthma (18, 71–74). Despite the discrepancies between studies on the important microbial features potentially associated with asthma, they have consistently shown that gut microbiota status in infancy, more so than early childhood, is associated with an altered risk of asthma. Collectively, this evidence supports the existence of a “critical period” during which the gut microbiota shapes the infant immune landscape and thus susceptibility to asthma later in life (70, 75).

## Conclusions and future directions

Breastfeeding profoundly influences the infant gut microbiota, and emerging evidence indicates that divergence from this evolutionarily conserved process can alter immune system maturation and influence asthma development. However, much remains to be discovered about the underlying mechanisms, including the prebiotic and probiotic properties of human milk. For example, we do not fully understand how HMOs and milk microbiota modulate the overall gut microbial community and whether this involves many or just a few key taxa. Moreover, there is tremendous variation in HMO utilization by different bacterial strains, and some HMOs are not utilized by the gut microbiota. The source and fate of bacteria in human milk is also uncertain. HMOs and milk microbiota may influence infant microbial communities at body sites other than the gut, including the airways and nasopharyngeal cavity, which may also play a role in asthma development. In addition to HMOs and microbiota, other milk components likely also impact the gut microbiota and/or the immune system, including cytokines, antimicrobial compounds, and immunoglobulins. These diverse components have largely been studied in isolation, yet they co-exist and interact in the mammary gland and the infant gut. New methods and integrated approaches will be required to disentangle the complex and dynamic interactions between HMOs, milk microbiota, and other milk components and their collective impact on the infant gut microbiota, immune function, and pediatric asthma. Understanding these processes will help define the role of breastfeeding and human milk in normal development, and could ultimately inform new microbiota-based strategies for asthma prevention.

## Author contributions

All authors contributed to the conception and design of this review and wrote sections of the manuscript. SM and MA compiled and integrated the individual sections. All authors contributed to manuscript revision, read and approved the submitted version.

### Conflict of interest statement

The authors declare that the research was conducted in the absence of any commercial or financial relationships that could be construed as a potential conflict of interest.
